# Annexin A2-Mediated Internalization of *Staphylococcus aureus* into Bovine Mammary Epithelial Cells Requires Its Interaction with Clumping Factor B

**DOI:** 10.3390/microorganisms9102090

**Published:** 2021-10-03

**Authors:** Yi-Tian Ying, Wei-Jia Ren, Xun Tan, Jing Yang, Rui Liu, Ai-Fang Du

**Affiliations:** 1 Department of Veterinary Medicine, Zhejiang University, Yuhangtang Road 866, Hangzhou 310058, China; yingyitian@zju.edu.cn (Y.-T.Y.); 22017103@zju.edu.cn (W.-J.R.); 21917098@zju.edu.cn (J.Y.); 3170100083@zju.edu.cn (R.L.); afdu@zju.edu.cn (A.-F.D.); 2 Veterinary Medical Center, Zhejiang University, Yuhangtang Road 866, Hangzhou 310058, China; 3 Institute of Preventive Veterinary Sciences, Zhejiang University, Yuhangtang Road 866, Hangzhou 310058, China

**Keywords:** *Staphylococcus aureus*, annexin A2, clumping factor B, mammary epithelial cells

## Abstract

Background: *Staphylococcus aureus* is a leading cause of contagious mastitis in dairy cattle. Internalization of *S. aureus* by bovine mammary gland epithelial cells is thought to be responsible for persistent and chronic intramammary infection, but the underlying mechanisms are not fully understood. Methods: In the present study, we evaluated the role of Annexin A2 (AnxA2), a membrane-binding protein, in *S. aureus* invasion into bovine mammary epithelial cell line (MAC-T). In vitro binding assays were performed to co-immunoprecipitate the binding proteins of AnxA2 in the lysates of *S. aureus*. Results: AnxA2 mediated the internalization but not adherence of *S. aureus*. Engagement of AnxA2 stimulated an integrin-linked protein kinase (ILK)/p38 MAPK cascade to induce *S. aureus* invasion. One of the AnxA2-precipitated proteins was identified as *S. aureus* clumping factor B (ClfB) through use of mass spectrometry. Direct binding of ClfB to AnxA2 was further confirmed by using a pull-down assay. Pre-incubation with recombinant ClfB protein enhanced *S. aureus* internalization, an effect that was specially blocked by anti-AnxA2 antibody. Conclusion: Our results demonstrate that binding of ClfB to AnxA2 has a function in promoting *S. aureus* internalization. Targeting the interaction of ClfB and AnxA2 may confer protection against *S. aureus* mastitis.

## 1. Introduction

*Staphylococcus aureus* is a major cause of infections in both human beings and a large number of animal species, including dairy cows. In humans, *S. aureus* causes a wide spectrum of diseases that range from cutaneous abscesses to life-threatening sepsis, accounting for considerable morbidity and mortality worldwide [1]. *S. aureus* is also a leading cause of contagious mastitis (infection of the mammary gland) in cattle and strikes the greatest challenge in the dairy industry [2,3,4,5]. *S. aureus* mastitis is extremely difficult to eradicate from herds, causing huge financial losses due to decreased milk yield, lowered milk quality, and premature culling of infected animals [2,6,7]. Furthermore, dairy products contaminated with toxins and enzymes produced by *S. aureus* can lead to food-borne diseases as it passes along the food chain [8,9]. In addition, cows have been recognized as the main animal reservoir for the emergent *S. aureus* clones that are epidemic in human populations [10,11]. 

As a facultative intracellular pathogen, *S. aureus* can be internalized by, and survive within, many types of non-phagocytic cells, such as human endothelial cells [12], epithelial cells [13,14], and keratinocyte [15]. Internalization of *S. aureus* by bovine mammary gland epithelial cells has also been demonstrated [16,17,18]. The internalization protects the bacteria from host immune defenses as well as antimicrobial killing, leading to the development of persistent or chronic infections [19,20,21]. Invasion of non-professional phagocytes by *S. aureus* is mediated by a number of adhesins on the bacterial surface, which interact either directly or indirectly with extracellular matrix (ECM) or specific surface receptors on the host cells [22]. MSCRAMMs (microbial surface component recognizing adhesive matrix molecules) are the largest family of adhesins, which target the host’s extracellular matrix proteins, such as collagen, fibrinogen, fibronectin (Fn), or laminin, for adhesion [23,24,25]. The interaction between Fn binding proteins (FnBPs, which are MSCRAMMs) on the bacteria and α5β1 integrin on the host cells via an Fn bridge has been considered as the most common pathway for the adhesion and internalization of *S. aureus* [26]. Bacterial engagement of the integrin α5β1 triggers intracellular signaling cascades and subsequent rearrangement of the host cell cytoskeleton to guide staphylococcal endocytosis [27]. Nevertheless, other studies have provided evidence for an FnBP-independent engulfment of *S. aureus* [16,28]. 

There is increasing evidence showing that Annexin A2 (AnxA2), a member of the annexin family of Ca^2+^-regulated phospholipid-binding and membrane-binding proteins, functions as a host receptor for bacterial adhesion and/ or invasion into non-phagocytic cells [29,30,31]. AnxA2 is expressed in the majority of cells and tissues and binds to numerous ligands, and is associated with multiple membrane related functions, including exocytosis and endocytosis, membrane domain organization, actin remodeling, and signal transduction [32]. More recently, an interaction between *S. aureus* clumping factor A (ClfA) and AnxA2 on bovine mammary cells has been identified in an in vitro protein binding assay [33]. However, the intracellular events initiated by bacterial engagement of AnxA2 are poorly understood. In addition, further evidence regarding the interaction of ClfA and AnxA2 is lacking. 

In the present study, we evaluated the engagement of AnxA2 in bacterial internalization into bovine mammary epithelial cells by using a clinical isolate of *S. aureus* (Clin-SA). In some experiments, a reference strain ATCC 25923 was used to validate the findings from Clin-SA. We demonstrate that bacterial engagement of AnxA2 regulates F-actin rearrangement via integrin-linked protein kinase (ILK)/p38 MAPK pathway. We have also examined Clin-SA lysates for the presence of AnxA2-binding proteins. Surprisingly, we identify ClfB but not ClfA as a component of the AnxA2-binding complex. Subsequently, we show that pretreatment of a recombinant ClfB protein enhances subsequent bacterial internalization, an effect that could be blocked by anti-AnxA2. Taken together, this work identifies ClfB as a novel AnxA2-binding protein that regulates the invasion of *S. aureus*.

## 2. Materials and Methods

### 2.1. Bacterial Strains and Culture Conditions

*S. aureus* strain (Clin-SA) was isolated from mastitic milk samples. The identity of the bacteria strain was confirmed by means of culture, Gram staining, and 16S rRNA gene sequencing. *S. aureus* strain ATCC 25923 was a gift from H-Q. Li, Lanzhou Institute of Animal Husbandry and Veterinary Pharmaceutical, Chinese Academy of Agricultural Sciences. Prior to use, bacteria were grown at 37 °C in brain heart infusion (BHI) with constant aeration (220 rpm) to establish the early exponential phase cultures (OD600 = 0.8 − 1.0). *S. aureus* colony-forming units (CFU) were quantified on Lysogeny broth (LB) plates incubated overnight at 37 °C. In some experiments, staphylococci were labeled with FITC prior to infection [19]. Labeled bacteria were extensively washed with Dulbecco’s phosphate-buffered saline (DPBS) prior to use.

### 2.2. Invasion and Adherence Assays 

Bovine mammary epithelial cell line MAC-T (generously provided from Zhu Y., Chinese Agricultural University, Beijing) and porcine intestinal epithelial cell line IPEC-J2 were cultured in Dulbecco’s Modified Eagle Medium (DMEM) supplemented with 10% fetal bovine serum (FBS) at 37 °C, 5% CO_2_. Cells were subcultured every 2 days. Bacterial invasion was evaluated with gentamicin protection assay [13]. In brief, MAC-T cells (unless stated otherwise) were seeded in 24-well tissue culture plates at 5 × 10^4^ cells/well and grown in growth medium. For confocal microscopy, glass coverslips were placed into the wells. After 24 h of incubation, the culture medium was aspirated and replaced with invasion medium (growth medium without FBS) and the cells infected with Clin-SA or ATCC 25923 at indicated multiplicity of infection (MOI) for 1 h. After washing with DPBS, invasion medium containing 0.2 μg/mL gentamicin was added into each well to kill all remaining extracellular *S. aureus*. Thereafter, the cells were washed twice with DPBS and intracellular bacteria released by incubation in 0.25% Triton X-100 for 25 min. Samples were serially diluted in DPBS and plated on LB agar plates for determination of the recovered colony forming units (CFU). An adherence assay was performed as described for the gentamicin protection assay, except that no antibiotic was added to the MAC-T monolayers before cell lysing. It is believed that the contribution of internalized bacteria to adherence is very low [34]. 

### 2.3. Cytochalasin D Assay

Cytochalasin D (Cyt D) assay procedure was carried out as described previously [13]. Briefly, MAC-T cells were preincubated with actin-depolymerization agent Cyt D (Sigma-Aldrich, Saint Louis, MO, USA) at 50 µg/mL 2 h prior to inoculation with bacteria to allow the disruption of the actin filaments and the inhibition of actin polymerization. After incubation with bacteria for 1 h at 37 °C, a gentamicin protection assay was performed as described above. 

### 2.4. Transmission Electron Microscopy

MAC-T cultures were grown in 6-well plates and infected with Clin-SA as described above. Following washing in the DPBS, the cells were fixed in 2.5% glutaraldehyde, postfixed in 1% OsO4, and processed for transmission electron microscopy (JEM-1200, JEOL, Japan) as previously described [18]. 

### 2.5. Confocal Microscopy

MAC-T cells were fixed with 4% paraformaldehyde for 30 min, followed by permeabilization in 0.25% Triton X-100 for 15 min. The cells were then incubated with 50 μM TRITC-labelled phalloidin (Sigma-Aldrich, Saint Louis, MO, USA) in invasion medium for 30 min at room temperature to visualize the actin cytoskeleton. The samples were viewed with a confocal laser scanning microscope (Olympus IX81-FV1000).

### 2.6. Recombinant ClfB Protein

To generate recombinant ClfB protein, a full-length *ClfB* DNA fragment was amplified by PCR by using ATCC 25923 strain chromosomal DNA as template and subcloned into expressing vector pGEX-4T-1 (Clontech, Mountain View, CA, USA). PCR primers were as follows: forward 5′-GGT ACC ACA TCA GTA ATA GTA GG-3′, reverse 5′-TCT TTA TGA TCT TGC TTG CGT T-3′. The recombinant GST-tag ClfB protein was produced in BL21 bacteria strain, purified by using BeyoGold™ GST-tag Purification Resin (Beyotime Biotech, Nanjing, China) according to the manufacturer’s protocol, and dialyzed at 4 °C by using a dialysis bag with molecular weight cut-off of 8–14 kDa (Sigma-Aldrich, Saint Louis, MO, USA). The dialyzed protein was stored at −80 °C prior to use.

### 2.7. Gene Silencing by siRNAs

Small interfering RNAs (siRNAs) targeting bovine *AnxA2* and *ILK* or negative control siRNA (NC-siRNA) were purchased from GenePharma (Shanghai, China). Cells were transfected with siRNAs using Lipofectamine 2000 (Invitrogen, Carlsbad, CA, USA) according to the manufacturer’s instructions. The siRNAs designed to target the coding regions of AnxA2 and ILK were as follows: *AnxA2*, 5′-GCG GGA UGC UCU GAA CAU UTT-3′ (sense), 5′-AAU GUU CAG AGC AUC CCG CTT-3′ (antisense); *ILK*, 5′-GCU ACA UGA AGG CAC CAA UTT-3′ (sense), 5′-AUU GGU GCC UUC AUG UAG CTT-3′ (antisense). Negative control siRNA sequences were 5’-UUC UCC GAA CGU GUC ACG UTT-3’ (sense) and 5’-ACG UGA CAC GUU CGG AGA ATT-3’ (antisense). The final concentration of the siRNAs was 20 nmol/L. Knockdown efficiencies were determined by qPCR and Western blot after 48 or 72 h of transfection.

### 2.8. Overexpression of AnxA2 in MAC-T

Expression plasmid coding the full length of the open reading frame (ORF) of *AnxA2* (Gene ID: 282689) was constructed by using pEGFP-C3 vector (Clontech, Mountain View, CA, USA). A Kozak sequence (GCCACC) was introduced immediately after the ATG to optimize the expression of the fusion protein. Cells were seeded at 2 × 10^5^ cells/well in 6-well cell culture plates and cultured overnight prior to transfection. Plasmid transfection was carried out using Lipofectamine^®^ 2000 reagent (Invitrogen, Carlsbad, CA, USA) according to the manufacture’s procedure. Cells transfected with empty plasmid were served as controls. After 48 h of transfection, the cells were subjected to bacterial infection at indicated MOI for 1 h. 

### 2.9. Western Blotting Analysis

Total cell lysates and immunoprecipitates were separated by sodium dodecyl sulfate (SDS)-12% polyacrylamide gel electrophoresis (PAGE) and blotted onto to polyvinylidene difluoride (PVDF) membranes (Millipore, USA). The following primary antibodies were used for immunodetection: AnxA2, ILK, and p-p38 (Santa Cruz Biotechnology) at 1:1000- to 1:2000-fold dilutions. After incubation overnight at 4 °C, blots were exposed to the appropriate horseradish peroxidase (HRP)-conjugated secondary antibody (Huabio, Hangzhou, China). Immunoreactive bands were visualized by electrochemiluminescent (ECL) (FDbio Science, Hangzhou, China). β-actin served as loading controls.

### 2.10. Immunoprecipitation and MS Analysis

Identification of the binding partners of host AnxA2 in the Clin-SA was carried out by co-immunoprecipitation (co-IP) coupled to mass spectrometry (MS) analysis. Bacteria were harvested at OD600 of 0.6–0.8 by centrifugation at 5000× *g* at room temperature for 5 min. The pellet was washed twice with DPBS, resuspended in a lysis buffer (50 mM Tris-HCl at pH8.9, 250 mM NaCl, 1 mM PMSF and 100 mg/mL lysozyme) at 1 g wet pellet/10 mL, and sonicated with a 150 Watt ultrasonic processor at 60% amplitude with pulse durations of 2 s on and 4 s off. The samples were centrifuged at 14,000× *g* at 4 °C for 30 min to remove the cellular debris and aggregate. The supernatant was used for subsequent binding analyses. Total protein concentrations were quantified by a BCA kit according to the manufacturer’s instruction (Meilunbio, Dalian, China). MAC-T cells were lysed with 1% Triton X-100 containing protease inhibitor cocktail on ice for 1 h. Thereafter, 400 μg protein from MAC-T cells was mixed with 500 μg protein from bacteria and the mixture was incubated overnight at 4 °C. The protein A/G magnetic beads (Bimake, Shanghai, China) conjugated with anti-AnxA2 mAb was added into the mixture to capture the AnxA2-binding proteins. The immunoprecipitates were then analyzed by SDS-PAGE, using 12% polyacrylamide gel, and detected by silver staining. The gel bands were excised and in-gel digested with 12.5 ng/μL trypsin in 25 mM NH_4_HCO_3_. LC–MS/MS analysis was performed on a Q Exactive mass spectrometer (Thermo Scientific, Shanghai, China) coupled to Easy nLC (Proxeon Biosystems, Odense, Denmark). The mass spectrometer was operated in positive ion mode. MS data were acquired using a data-dependent top 10 method, dynamically choosing the most abundant precursor ions from the survey scan (300–1800 m/z) for higher energy collisional dissociation (HCD) fragmentation. Full MS AGC target was 3E6 with a maximum injection time of 10 ms. Dynamic exclusion duration was 40.0 s. Survey scans were acquired at a resolution of 70,000 at *m*/*z* 200 and resolution for HCD spectra was set to 17,500 at m/z 200, and isolation width was 2 *m*/*z*. Normalized collision energy was 30 eV and the underfill ratio, which specifies the minimum percentage of the target value likely to be reached at maximum fill time, was defined as 0.1%. The instrument was run with peptide recognition mode enabled. The MS/MS spectra were searched using MASCOT engine (Matrix Science, London, UK; version 2.4) against the UniProtKB *S. aureus* database (105867 total entries, downloaded 14 June 2019). Search parameters were set as follows: enzyme, Trypsin; peptide mass tolerance, 20 ppm; fragment ion mass tolerance, 0.1 Da; maximum missed cleavages allowed, 2; fixed modification, carbamidomethyl (C); variable modification, oxidation (M); and ion score >20. 

### 2.11. Pull-Down Assay

An in vitro pull down assay was performed using BeyoGold™ GST-tag Purification Resin (Beyotime Biotech, Nanjing, China) according to the manufacturer’s instructions. In brief, GST or GST-ClfB was incubated overnight with MAC-T cell lysates and Sepharose CL-6B beads in NP-40 buffer at 4 °C. After washing in NP-40 buffer 3 times, the beads were suspended in SDS-PAGE loading buffer and boiled for 5 min. The protein complex was separated by SDS-PAGE and then analyzed by Western blot.

### 2.12. Statistical Analysis

Data are presented as mean ± standard deviation (s.d.). Statistical analysis was performed with the SPSS software v.22. The differences between two groups were analyzed using Student’s *t*-test, while one-way ANOVA followed by LSD correction was applied to compare more than two groups. Differences were considered significant for *p* <  0.05. 

## 3. Results

### 3.1. AnxA2 Is Required for the Internalization of S. aureus by MAC-T Cells

**Figure 1:**
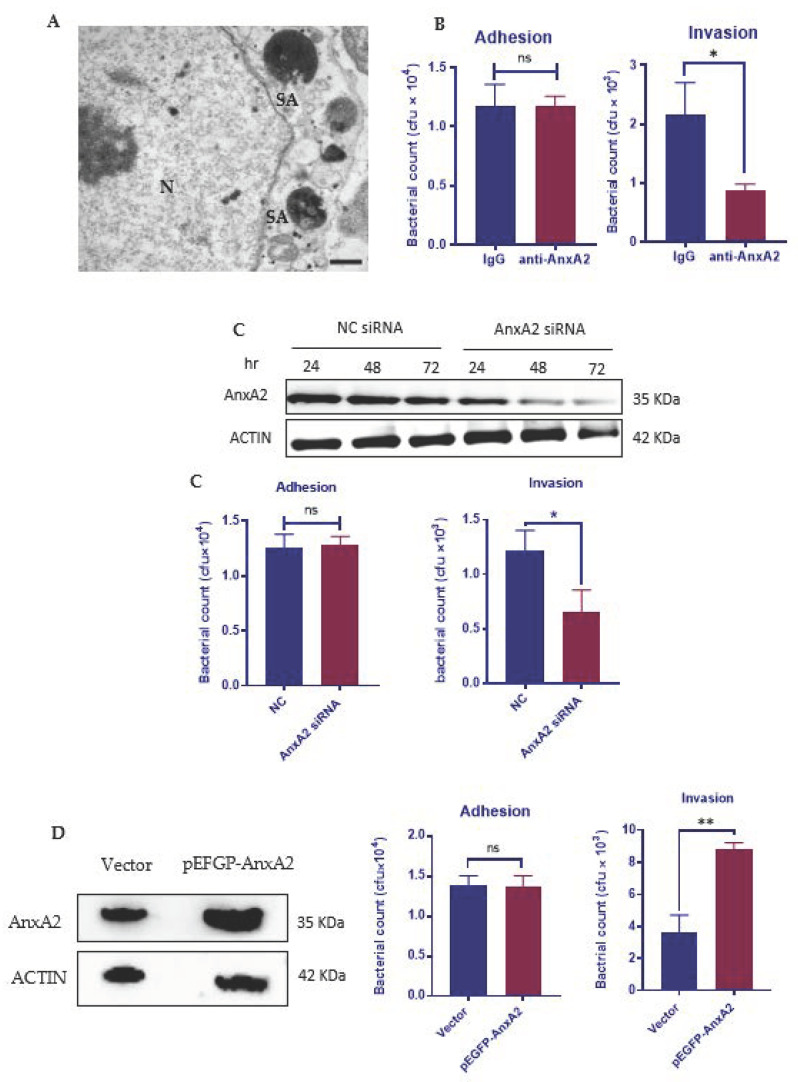
AnxA2 mediates the invasion of *S. aureus*. (**A**) Transmission electron micrograph showing internalization of a clinical isolate of *S. aureus* (Clin-SA) into MAC-T. Two engulfed bacteria (boxed) are displayed at high magnification. Photograph represents a 1 h co-culture of cell monolayer with Clin-SA at a MOI of 50. N: nucleus; C: cytoplasm. Scale bar: 500 nm. (**B**–**D**) Effect of AnxA2 on *S. aureus* adhesion to and uptake by MAC-T cells. MAC-T cells were pre-incubated with anti-AnxA2 antibody (17.5 μg/mL) or human IgG (17.5 μg/mL) for 2 h to block AnxA2 function (**B**), treated with AnxA2 siRNA for 72 h to knock down the expression of AnxA2 (**C**), or transfected with a pEGFP-AnxA2 plasmid for 48 h to overexpress AnxA2 (**D**) prior to Clin-SA infection (MOI: 50). The adhesion and invasion assays were performed after incubation of MAC-T with bacteria for 1 h. Dates are mean values ± s.d. of three replicates. * *p* < 0.05; ** *p* < 0.01; ns: no significant difference.

*S. aureus* was able to invade into bovine mammary epithelial cells (Figure 1A) [18]. To clarify whether AnxA2 has a functional role in the adherence or invasion of *S. aureus*, or both, we blocked the membrane function of AnxA2 by incubating MAC-T cells with an anti-AnxA2 antibody. Interestingly, blocking AnxA2 function at the cell surface had no effect on *S. aureus* adherence, but led to a significant decrease in the intracellular bacteria protected from gentamicin killing (Figure 1B). Further studies were conducted to evaluate the role of host AnxA2 on *S. aureus* adherence and internalization by using AnxA2-depleted MAC-T cells. Western blotting of whole extracts from cells treated with siRNA targeting AnxA2 for 72 h revealed marked depletion of AnxA2 (Figure 1C). While AnxA2 knockdown resulted in a significant decrease in *S. aureus* uptake, we did not observe a significant change in *S. aureus* adherence (Figure 1C). To further confirm the role of AnxA2, we performed overexpression of AnxA2 in MAC-T cells (Figure 1D). AnxA2 overexpression significantly enhanced bacterial invasion without altering the number of bacteria attached to MAC-T cells (Figure 1D). Taken together, our observations suggest that AnxA2 is necessary for staphylococci internalization but not adherence.

### 3.2. AnxA2 Contributes to S. aureus-Induced Actin Cytoskeleton Reorganization

**Figure 2:**
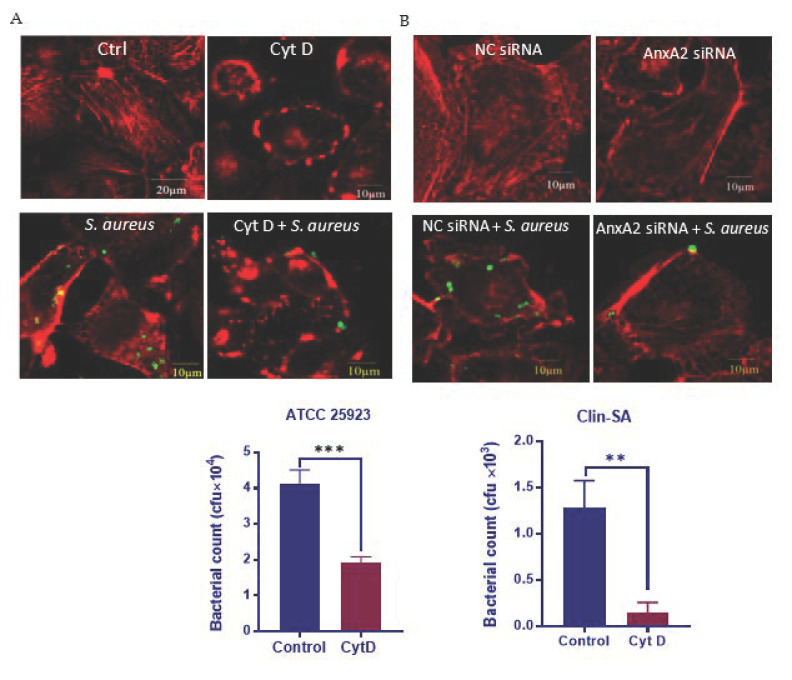
Engagement of AnxA2 in *S. aureus*-induced cytoskeleton reorganization. MAC-T cells were pre-treated with cytochalasin D (Cyt D) for 2 h (**A**–**C**) or AnxA2 siRNAfor 72 h (**D**), followed by infection with clinical isolate of *S. aureus* (Clin-SA, FITC-labeled) or *S. aureus* strain ATCC 25923 for 1 h. F-actin was visualized by TRITC-conjugated phalloidin (**A**,**D**). Representative confocal microscopy images of cells infected with Clin-SA are shown (Green: *S. aureus*; Red: F-actin). Invasion of Clin-SA (**B**) as well as ATCC 25923 (**C**) was determined by gentamicin protection assay. The graph shows mean values ± s.d. of three replicates. Each assay was repeated at least three times. ** *p* < 0.01 and *** *p* < 0.001.

Actin cytoskeleton is crucial for *S. aureus* uptake by MAC-T, as Cyt D, which interferes with F-actin polymerization (Figure 2A), severely blocks Clin-SA internalization into MAC-T (Figure 2A,B) [27,35]. Similarly, Cyt D treatment led to a significant reduction in the uptake of a reference strain of *S. aureus*, ATCC 25923, by MAC-T cells (Figure 2C), suggesting the cytoskeleton-dependent invasion of *S. aureus* is not strain-specific. Since AnxA2 functions in the dynamic reorganization of F-actin cytoskeleton [36], we next used AnxA2-deficient MAC-T cells to evaluate the role of AnxA2 in *S. aureus*-induced cytoskeleton reorganization. As expected, AnxA2 depletion led to a major change in the organization of actin cytoskeleton upon *S. aureus* infection (Figure 2D). This result, along with the finding that loss of AnxA2 caused a significant reduction in bacterial invasion (see above, Figure 1C), indicates that AnxA2 plays a role in *S. aureus*-induced reorganization of actin cytoskeleton.

### 3.3. Bacterial Engagement of AnxA2 Activates ILK/p38 MAPK Pathway 

**Figure 3:**
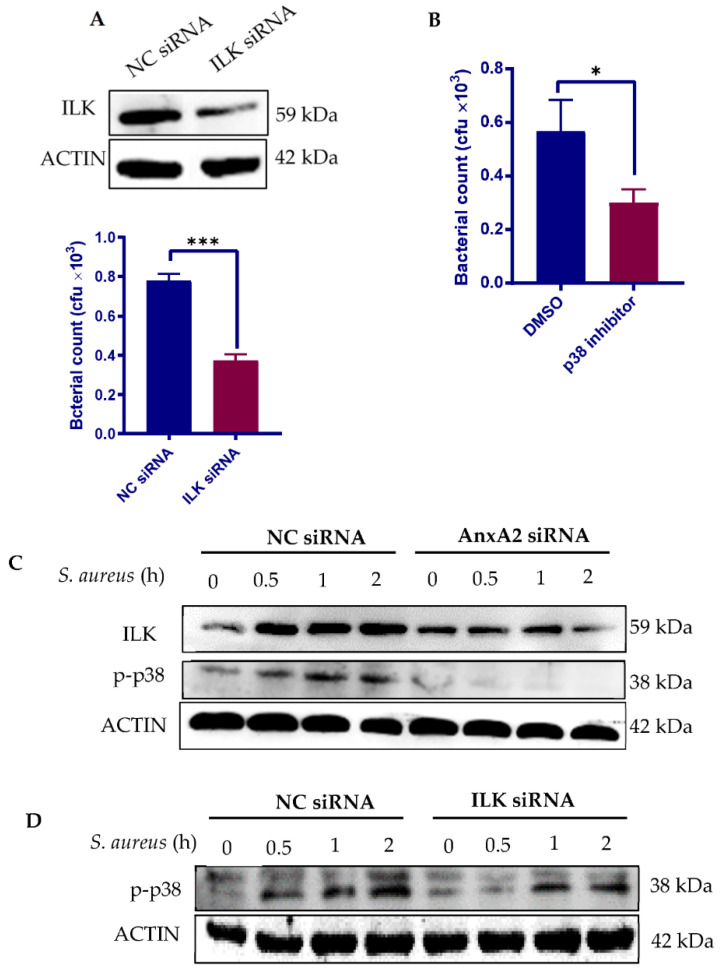
AnxA2 engagement activates ILK/p38 MAPK pathway. (A) Evaluation of the effect of ILK on *S. aureus* internalization by MAC-T. MAC-T cells were treated with either NC siRNA or ILK siRNA for 72 h. Whole-cell extracts were analyzed by immunoblotting with anti-ILK (upper panel). β-actin is shown as a loading control. The ILK-deficient cells were then co-cultured with clinical isolate of *S. aureus* (Clin-SA) at an MOI of 50 for 1 h. Bacterial invasion was determined by gentamicin protection assays (lower panel). The graph shows mean values ± s.d. of three replicates. (**B**) Evaluation of the effect of p38 MAPK on *S. aureus* internalization by MAC-T. MAC-T cells were pre-treated with SB203580, a selective inhibitor of p38 MAPK, at 10 μM for 2 h and infected with Clin-SA at an MOI of 50 for 1 h. Bacterial invasion was determined by gentamicin protection assays. The graph shows mean values ± s.d. of three replicates. (**C**) Evaluation of the effect of AnxA2 engagement on ILK expression and p38 MAPK phosphorylation. MAC-T cells were treated with either NC siRNA or AnxA2 siRNA for 72 h and then subjected to Clin-SA infection at a MOI of 25 for 2 h. Whole-cell extracts were analyzed by immunoblotting with antibodies against AnxA2, ILK and phosphorylated p38 MAPK. β-actin is shown as a loading control. (**D**) Evaluation of the effect of ILK engagement on p38 MAPK phosphorylation. MAC-T cells were treated with siRNA and Clin-SA as described in (**A**). Whole-cell extracts were analyzed by immunoblotting with antibodies against ILK and phosphorylated p38 MAPK. β-actin is shown as a loading control. * *p* < 0.05; *** *p* < 0.001.

We next sought to determine the downstream signal molecules involved in AnxA2-mediated *S. aureus* invasion. We focused on ILK, which is essential for *S. aureus* internalization [37,38], and p38 mitogen-activated protein kinase (MAPK), a member of MAPK family that plays a role in regulating F-actin reorganization [39]. As expected, depletion of ILK with siRNA (Figure 3A) or inhibition of p38 MAPK activity (Figure 3B) interfered with *S. aureus* invasion into MAC-T cells. To verify whether AnxA2 engagement leads to upregulation in ILK and p38 MAPK activation, the expression of AnxA2 in MAC-T was knocked down before *S. aureus* infection. Western blot analysis showed that *S. aureus* infection increased the levels of ILK protein as well as p38 MAPK phosphorylation in a time-dependent manner in cells treated with negative control (NC) siRNA (Figure 3C). On the contrary, loss of AnxA2 significantly reduced the ILK response to *S. aureus*, and dramatically blocked *S. aureus*-induced p38 MAPK phosphorylation (Figure 3C), indicating that both ILK and p38 MAPK act downstream of AnxA2. Signaling from ILK has been shown to activate p38 MAPK [40,41]. Using ILK-deficient MAC-T cells, we confirmed that ILK is critical for *S. aureus*-induced p38 MAPK phosphorylation (Figure 3D). Taken together, our results suggest that ILK/p38 signaling pathway drives the AnxA2-mediated invasion of staphylococci.

### 3.4. AnxA2 Is a Binding Partner of S. aureus ClfB 

**Figure 4:**
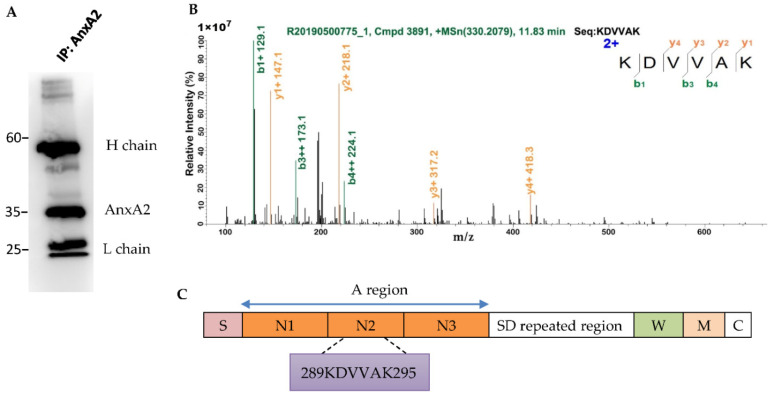
Identification of *S. aureus* ClfB as a binding partner of AnxA2. (**A**) AnxA2 detected by Western blot. Whole lysates from MAC-T cells were incubated overnight at 4 °C with the extracts from clinical isolate of *S. aureus* (Clin-SA) grown to early exponential phase. The protein A/G magnetic beads conjugated with anti-AnxA2 mAb were used to capture AnxA2-binding complex. Thereafter, protein complexes were separated on 12% SDS-PAGE and subjected to Western blot analysis. IP, immunoprecipitation; H chain, IgG heavy chain; L chain, IgG light chain. (**B**) Data analysis of the MS/MS spectrum led to identification of a peptide with the sequence KDVVAK, which was part of ClfB protein. b- and y-ions are labelled. (**C**) Schematic diagram of ClfB structure indicating the signal sequences (S), the N-terminal ligand binding A region with three subdomains, N1, N2, and N3, the Ser–Asp dipepide (SD) repeated region, the wall-spanning regions (W), the membrane-spanning regions (M), and the charged C-terminal tail (C). The location of peptide sequence of ClfB recognized by AnxA2 is also shown.

**Figure 5:**
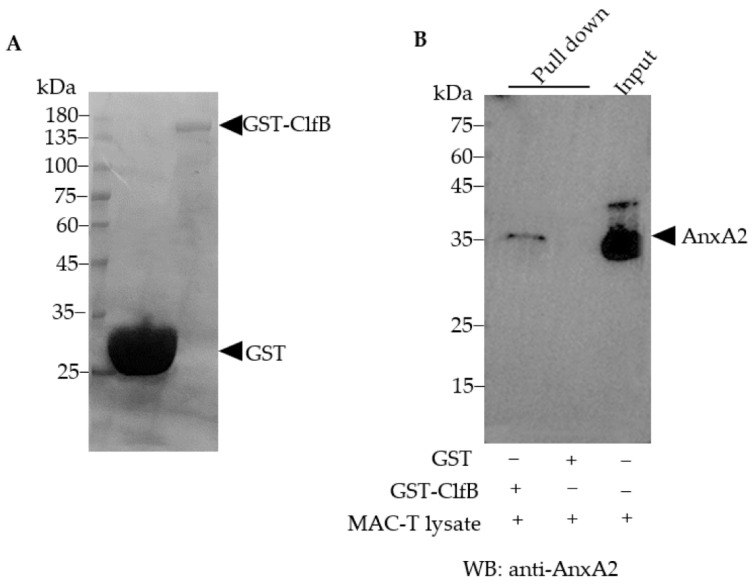
Binding of *S. aureus* ClfB to AnxA2 of MAC-T. (**A**) GST and GST-tagged recombinant ClfB produced from *E. coli* BL21 were purified and assessed by SDS-PAGE, and the proteins were visualized by Coomassie brilliant blue staining. (**B**) GST-ClfB protein and GST protein were combined to Sepharose CL-6B beads, and incubated with whole cell lysates prepared from MAC-T. The protein complexes were released by boiling, electrophoresed on 12% SDS-PAGE, and subjected to Western blot analysis with anti-AnxA2 antibody.

Having established the importance of host AnxA2 in *S. aureus* invasion, we next sought to determine its interacting proteins in bacteria. Because only a limited number of bacteria were internalized by MAC-T cells, we were restricted in our studies to use the whole lysates of *S. aureus*-infected MAC-T to determine the AnxA2 interactors from intracellular bacteria by using co-IP assay. In order to mimic in vivo condition, the lysates from MAC-T cells were incubated with *S. aureus* extracts. The AnxA2-binding complex was then captured by Protein A/G Beads conjugated with anti-AnxA2 mAb. The presence of AnxA2 in the protein complex was confirmed by silver stain (data not shown) and Western blot analysis (Figure 4A) after the immunoprecipitated proteins were separated by SDS-PAGE. Using LC–MS/MS analysis, we identified 299 known entries of *S. aureus* in the immunoprecipitates. Among these, *S. aureus* ClfB was identified as a candidate of AnxA2-binding protein (Table S1). Fragment spectrum of a peptide derived from ClfB (G0XY85_STAAU) is shown in Figure 4B. The sequence recognized by AnxA2 is located at the N-terminal ligand binding A region of ClfB (Figure 4C). Notably, ClfA was not included in the bound proteins of AnxA2 (Table S1). In order to confirm the physical interaction of ClfB and AnxA2, a pull-down assay was performed by using GST-fused ClfB as bait protein. Because the genome of the Clin-SA was not sequenced in this work, we therefore amplified *ClfB* gene from ATCC 25923 to produce recombinant ClfB protein. Western blot analysis demonstrated the presence of AnxA2 in the protein complexes obtained from the GST pull-down assay (Figure 5).

### 3.5. Interaction of ClfB and AnxA2 Regulates the Internalization of S. aureus

**Figure 6:**
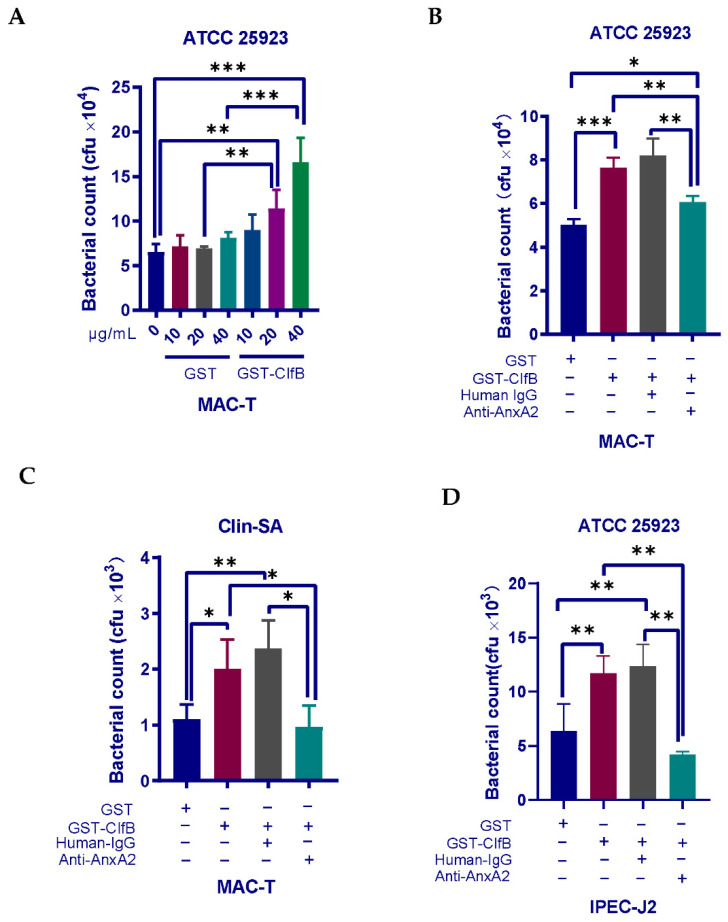
Interaction of ClfB and AnxA2 is required for *S. aureus* invasion. (A) Recombinant ClfB enhances *S. aureus* invasion in a dose-dependent manner. MAC-T cells in 24-well plates were pretreated with recombinant or GST-tagged ClfB (0–40 μg/mL) for 1 h, followed by co-incubation with *S. aureus* ATCC 25923 at an MOI of 25 for another 1 h. (**B**–**D**) Anti-AnxA2 blocks recombinant ClfB –induced *S. aureus* internalization. MAC-T (B and C) or IPEC-J2 (**D**) were treated with anti-AnxA2 antibody (17.5 μg/mL) or human IgG (17.5 μg/mL) for 2 h prior to exposure to GST-tagged recombinant ClfB (20 μg/mL) for 1 h. After washing in DPBS 3 times, cells were co-cultured with *S. aureus* ATCC 25923 (B and D) at an MOI of 25 or clinical isolate of *S. aureus* (Clin-SA) (**C**) at an MOI of 50 for 1 h. Cells treated with recombinant GST (20 μg/mL) or GST-tagged recombinant ClfB (20 μg/mL) alone were served as controls. Bacterial invasion was determined by gentamicin protection assays. The graph shows mean values ± s.d. of three replicates. * *p* < 0.05; ** *p* < 0.01; and *** *p* < 0.001.

The importance of AnxA2 binding by ClfB for *S. aureus* invasion was evaluated. We first incubated MAC-T cells with a recombinant ClfB protein prior to *S. aureus* ATCC 25923 infection. If ClfB merely severs as an adhesion molecule mediating the attachment of *S. aureus* to host cells, one could expect a reduction in *S. aureus* invasion when ClfB’s ligands on host cells are occupied by recombinant ClfB protein. In sharp contrast to our expectation, gentamicin protection assays showed that pre-treatment with ClfB protein increased *S. aureus* uptake by MAC-T cells in a dose-dependent manner (Figure 6A), suggesting that ClfB plays a more significant role in bacterial invasion than in adherence. Since we have determined an interaction of AnxA2 and ClfB in our protein–protein binding assays, we next asked whether ClfB-mediated *S. aureus* internalization is dependent on AnxA2. To this end, we incubated the cells with an anti-AnxA2 antibody for 2 h before the addition of recombinant ClfB protein. As expected, pretreatment with anti-AnxA2 blocked the effect of recombinant ClfB protein on ATCC 25923 invasion into MAC-T cells, while no significant effect of the negative control IgG (human IgG) on *S. aureus* ATCC 25923 invasion was observed (Figure 6B). Similar results were obtained when using Clin-SA strain for infection (Figure 6C). In order to test if this response is specific to bovine mammary epithelial cells, we also used porcine intestinal epithelial cell line (IPEC-J2) for experiments. In line with the findings on MAC-T, pretreatment of anti-AnxA2 also blocked ClfB-stimulated increase in *S. aureus* invasion into IPEC-J2 (Figure 6D). Taken together, our results indicate that interaction of ClfB and AnxA2 is required for *S. aureus* invasion.

## 4. Discussion

In the present study, we demonstrate that AnxA2 is critical for *S. aureus* internalization into bovine mammary epithelial cells, and that ILK/p38 MAPK signal pathway is involved in this process. In addition, we identified *S. aureus* ClfB as a binding partner of AnxA2. Our results show that the interaction of AnxA2 and ClfB is necessary for *S. aureus* internalization. Although both host AnxA2 [31] and *S. aureus* ClfB [42] have been implicated in *S. aureus* infection, ours is the first to reveal a direct interaction of AnxA2 and ClfB.

AnxA2 has been shown to mediate the uptake of a wide range of bacterial species by nonprofessional phagocytic cells, for example *Pseudomonas aeruginosa* [30], *Salmonella typhimurium* [43], and *Mycoplasma pneumoniae* [29]. Early studies suggest that AnxA2 assists *S. aureus* adhesion [31]. Our observations, however, suggested that AnxA2 engagement induced bacterial invasion rather than adherence. The uptake of bacterial pathogens by non-professional phagocytic cells requires not only interaction with cell surface proteins but also subversion of actin cytoskeletal dynamics. AnxA2 is well known for its F-actin binding and bundling activity in the dynamic remolding of the actin cytoskeleton [44,45,46,47,48]. Thus, the underlying mechanism by which AnxA2 triggers invasion appears to be associated with its ability to regulate actin cytoskeleton. Supporting this hypothesis, we observed that AnxA2 depletion prevented *S. aureus* invasion, concomitant with disruption of F-actin reorganization. 

The signaling pathways downstream of AnxA2 during *S. aureus* invasion remain largely unknown. By using AnxA2-deficient MAC-T cells, we demonstrated that AnxA2 engagement is required for *S. aureus*-stimulated upregulation in ILK, a serine-threonine protein kinase that binds the cytoplasmic domains of β1 and β3 integrin subunits to regulate integrin-actin connection and acts as the immediate downstream effector of integrin signal transduction [49,50]. Given that integrin α5β1 promotes bacterial adhesion and triggers uptake process [35], our results allow us to argue that there exists a functional link between AnxA2 and integrin α5β1 during *S. aureus* internalization. Indeed, AnxA2 has been shown to activate integrin α5β1 via direct protein–protein interaction between AnxA2 and integrin α5 cytoplasmic domain [51]. In another study, AnxA2 has been shown to mediate the internalization of cell surface integrin β1 [52]. However, further investigation is required to evaluate the potential associations between AnxA2 and integrins during *S. aureus* infection. We have also identified p38 MAPK as a downstream effector of ILK, in line with previous studies demonstrating that ILK regulates actin cytoskeleton remodeling via p38 MAPK phosphorylation [53]. Taken together, our results suggest that AnxA2 couples to ILK/p38 MAPK pathway for modulating *S. aureus* invasion.

*S. aureus* expresses various surface proteins that mediate the adhesion and invasion of the pathogen into host cells by binding to different host molecules. We next carried out in vitro protein-binding assays to identify the AnxA2 binding proteins on the bacteria. One of our objectives is to confirm the interaction of ClfA and AnxA2, which has been previously reported by Ashraf et al. [33]. Surprisingly, ClfA protein was not detectable in the AnxA2-precipitated proteins. Indeed, our work identified AnxA2 as the ligand recognized by ClfB. It should be noted that, in the study of Ashraf et al. [33], a recombinant ClfA protein was used as a bait to pull down lysates of bovine mammary epithelial cells. By this means, ClfB was not included in the in vitro binding system. In contrast, in the present study, we designed a protein–protein binding assay to determine the interactors of AnxA2 in bacteria lysates, which enabled both ClfA and ClfB proteins to be exposed to AnxA2 simultaneously. It is also noteworthy that we used bacteria in the early exponential phase of growth for our in vitro binding assay. Early studies have shown that ClfA protein is expressed throughout bacterial growth, whereas ClfB is only expressed in the early exponential phase of growth [54,55]. Therefore, our finding that AnxA2 binding to ClfB rather than ClfA raises a possibility that ClfB has higher binding ability to AnxA2 than ClfA, or the expression of ClfB is higher than ClfA in early exponential phase, or both. Nevertheless, our work does not exclude the possibility that ClfA could bind to AnxA2 when cells lose ClfB expression in late exponential or stationary phase. 

ClfB has been previously shown to promote the adherence of *S. aureus* to host cells [42,54]. Based on these findings, we hypothesized that pre-occupation of ClfB ligands on MAC-T using recombinant ClfB protein could lead to reduced *S. aureus* adherence, resulting in decreased bacterial internalization. However, counter to our initial prediction, we found that pre-treatment with recombinant ClfB protein enhanced subsequent *S. aureus* uptake by MAC-T in a dose-dependent manner. The result thus allows us to argue that ClfB functions to drive *S. aureus* internalization rather than adherence. In addition, we found that recombinant ClfB protein-induced *S. aureus* uptake by MAC-T and IPEC-J2 was specially blocked by anti-AnxA2 antibody, suggesting that binding of ClfB to AnxA2 is required for staphylococcal invasion into host cells.

The significance of ClfB in the context of *S. aureus* mastitis remains unclear. Notably, although both ClfB and ClfA have been implicated in the pathogenesis of *S. aureus* in various animal models [42,56,57,58,59], their contributions appear to be distinct, considering the fact that immunization with ClfA only provides modest protection [60] whereas vaccination with ClfB confers significant protection against *S. aureus* infection [56]. In addition, the frequency of *ClfB* gene was found to be higher than that of *ClfA* in *S. aureus* isolates for humans [61] and dairy cattle [62]. Additional research is thus warranted to define the relative contributions of these two proteins to the development of *S. aureus* mastitis in bovine.

## 5. Conclusions

In summary, the present study reveals a novel, previously unidentified role for ClfB-AnxA2 binding in *S. aureus* invasion. We also defined a signal transduction pathway downstream of AnxA2, which involves the upregulation of ILK and subsequent activation of p38 MAPK. We show for the first time that binding of ClfB to AnxA2 plays a role during *S. aureus* invasion into MAC-T. It is suggested that targeting the association between ClfB and AnxA2 might confer protection against *S. aureus* mastitis in dairy cattle.

## Data Availability

All data analyzed during this study are included in this published article and its supplementary information flies.

## Supplementary Materials

The following are available online at https://www.mdpi.com/article/10.3390/microorganisms9102090/s1, Table S1: Peptides identified by LC–MS/MS analysis of AnxA2-precipitated *S. aureus* proteins.
